# Multi-Manifold Learning Fault Diagnosis Method Based on Adaptive Domain Selection and Maximum Manifold Edge

**DOI:** 10.3390/s25175384

**Published:** 2025-09-01

**Authors:** Ling Zhao, Jiawei Ding, Pan Li, Xin Chi

**Affiliations:** School of Information Science and Engineering, Chongqing Jiaotong University, Chongqing 400074, China; 622-200070037@mails.cqjtu.edu.cn (J.D.); 631-607010505@mails.cqjtu.edu.cn (P.L.); 622-200070038@mails.cqjtu.edu.cn (X.C.)

**Keywords:** fault diagnosis, multi-manifold learning, adaptive neighborhood selection, feature fusion reduction, manifold edge distance

## Abstract

The vibration signal of rotating machinery is usually nonlinear and non-stationary, and the feature set has information redundancy. Therefore, a high-dimensional feature reduction method based on multi-manifold learning is proposed for rotating machinery fault diagnosis. Firstly, considering the non-uniformity of multi-fault feature distribution and the sensitivity of domain selection in traditional manifold learning methods, the neighborhood size of each data point is selected adaptively by using the relationship between neighborhood size and sample density. Then, the between-manifold graph and within-manifold graph are constructed adaptively by the class information, and the divergence matrix and edge distance corresponding to the manifold graph are calculated. Feature fusion reduction is achieved by maximizing edge distance and minimizing within-class differences. Finally, the multi-manifold theoretical dataset and several rotating machinery fault datasets are selected for testing. The results show that the proposed algorithm has higher fault identification accuracy than traditional manifold learning methods.

## 1. Introduction

In recent years, advanced sensor technology, data acquisition equipment, and computer storage equipment have enabled us to accumulate massive amounts of industrial big data. If all this multi-sensor big data can be processed and analyzed effectively, potential faults can be discovered early on, and reasonable operation and maintenance programs can be followed to ensure that rotating machinery operates safely [1]. However, vibration signals from rotating machinery are often characterized by both nonlinearity and non-stationarity, which makes them difficult to analyze using traditional methods. These signals exhibit time-varying statistical properties (e.g., mean, variance) and nonlinear relationships between the signal and mechanical component interactions. On the other hand, large-scale data analysis has introduced new challenges to the fault diagnosis of rotating machinery. For example, the process of data mining and pattern recognition using high-dimensional data is often plagued with problems such as “data explosion” and “dimension disaster”. Thus, mining valuable information accurately and efficiently has become a central problem in research related to rotating machinery fault diagnosis.

Feature reduction is an effective method to analyze and deal with the above-mentioned problems. Feature reduction essentially reduces the complexity and number of features in the original high-dimensional space to improve the performance of the data mining system. It simplifies the space by selecting redundant and unimportant features or combining the original feature changes into fewer features. Some scholars have explored many feature reduction methods [2].

Through manifold learning, researchers have been able to curate the nonlinear structure of original data in high-dimensional space and find the advantage of its intrinsic dimension [3,4,5]. Confronted with the nonlinear nature of most datasets, linear dimension reduction algorithms such as principal component analysis, linear discriminant analysis, and multidimensional scaling are poor for high-dimensional nonlinear data. Manifold learning has proven its effectiveness by unearthing prominent fault characteristics from the convolution of high-dimensional datasets; the most well-known ones are isometric feature mapping (ISOMAP), local linear embedding (LLE), and Laplacian Eigenmap [6,7,8]. Manifold learning-based feature reduction methods have revealed novel diagnostic methodologies which not only pare down feature dimensions but also sift out the extraneous ones [9].

But these methods assume that the data is distributed on the same manifold and use it as a modeling basis to ensure the internal structure remains unchanged when the data is mapped from high-dimensional space to a low-dimensional subspace. But previous studies have shown that rotating machinery has complex fault mechanisms, and not all faults exist in the same manifold which inevitably affects the further improvement of identification accuracy [10]. So multi-manifold learning methods have become the new direction for dimensionality reduction research on fault datasets.

Methods like Uniform Manifold Approximation and Projection for Dimension Reduction (UMAP) have been developed to prove strong diagnostic capabilities even in high noise conditions [11]. And the integration of discriminant local preservation projection with sparse autoencoder represents a leap in capturing both the macro- and microstructures within data [12].

Although these methods marked strides in classification, the critical task of discerning important differentiated information in classification still needs enhancement. The introduction of label information and refined similarity metrics, employing cosine distance for neighbor graph construction and label-centric weighting, has shown potential in more nuanced feature dimension reduction within high-dimensional spaces [13,14]. The challenge of constructing manifold neighbor graphs spurred further innovation, as seen with the local sparse discriminant preserving projection technique. This approach prioritizes relevant features and uses class labels to build comprehensive within and between-manifold graphs [15]. Similarly, the refinement of optimization functions to emphasize within-class and between-class variance accounts for multi-manifold relationships and has improved the fault feature extraction accuracy [16].

The scarcity of annotations in real datasets has prompted semi-supervised methodologies such as SDMM, which use graph-based multi-manifold modeling and convex optimization improvements for tasks like video dataset action recognition [17]. Statistical significance and the preservation of local data geometries address neighborhood parameter selection, and unsupervised subspace learning (USFN) added the concept of flexible neighborhoods, circumventing noise and neighborhood selection issues [18,19]. Furthermore, aided by adaptive neighborhood selection, algorithms like LTSA for adaptive sample point neighborhood selection enhance classification accuracy [20].

As commendable as these advancements are, they still have their vulnerabilities. The existing multi-manifold analysis methods only emphasize bringing similar samples closer and dissimilar samples farther away, but do not consider the aliasing problem that occurs between different manifolds. When different manifolds overlap or lie close to each other in high-dimensional space without being effectively separated, simply pulling features of different classes apart may not prevent boundary confusion. This often results in ambiguous class boundaries and increased distortion after dimensionality reduction. So, between-manifold modeling and selection of optimal neighborhood parameters for manifold learning remain elusive tasks. The upgrades are about choosing neighborhood parameters while preserving the integrity of data geometry, whilst simultaneously minimizing the impact of noise and parameter selection on the outcome.

Based on this, this paper proposes an ALLRMM model, which can adaptively adjust the neighborhood selection strategy based on local density, and count the multi-manifold edge margin, thereby dealing with the general situation that the data contains linear and nonlinear structures and overlaps with each other in real situations. Finally, it achieves the fault diagnosis of rotating machinery. Through this attempt, the integrity of data geometry can be preserved and the impact of noise and parameter selection minimized. The contributions of this paper are as follows:

(1) Most of the fault diagnosis methods for rolling bearings focus on the mean value of all samples or the mean value of a certain class of samples, while the description of local information is ignored. Therefore, based on modeling the multi-manifold subspace and using within-class and between-class differences, this paper innovatively applies the multi-manifold learning dimensionality reduction algorithm to realize the efficient classification of fault modes of rolling bearings.
(2) To solve the problem of sensitive parameter selection in the field of multi-manifold learning, the relationship between local sample density and neighborhood parameter values was tried, and the local neighborhood parameter size of each sample point was adjusted adaptively by defining the density scaling factor, so as to build the within- and between-manifold graphs adaptively. This helps to recognize boundaries between multiple manifolds, making the data samples more distinguishable.
(3) Two different types of datasets are selected for experimental analysis, and multiple nonlinear dimension reduction algorithms (KPCA, LLE, LLRMM, and UMAP) are compared to verify the proposed algorithm’s advantages in dimension reduction effect and recognition rate. The results show that using a manifold space for feature fusion reduction can lead to better classification.

## 2. Manifold Learning

Manifold learning is a class of nonlinear dimensionality reduction methods based on the core assumption that “high dimensional observed data often lie or approximately lie on a low dimensional smooth manifold embedded in the ambient space”, aiming to map the samples to a visual or compact low-dimensional representation while preserving the intrinsic geometry, topology, or statistical structure of the data. ISOMAP uses the geodesic distance calculated by Floyd-Warshall and achieves global isometric embedding through multidimensional scaling (MDS); Kernel Principal Component Analysis (Kernel PCA) implicitly maps the data to a high-dimensional reproducing kernel Hilbert space and then performs linear PCA, which can be regarded as the kernelized generalization of linear manifold learning; Local Tangent Space Alignment (LTSA) estimates the tangent space in each neighborhood first, and then globally aligns to minimize the tangent space projection error; Random Neighbor Embedding (SNE) and its improved version T-SNE map the high-dimensional neighborhood probabilities to low dimensions using Gaussian or Student-t distributions, minimizing the KL divergence through gradient descent, and are particularly excellent in visualization tasks; UMAP characterizes neighborhood relationships using fuzzy simplex complexes, combining Riemannian geometry and algebraic topology ideas, achieving fast speed and good global structure preservation in visualization and low-dimensional representation; LLE minimizes the linear reconstruction error of the neighborhood to maintain local linear relationships and then solves the sparse feature problem to obtain the low-dimensional coordinates. The most classic one is the LLE model.

**Figure 1:**
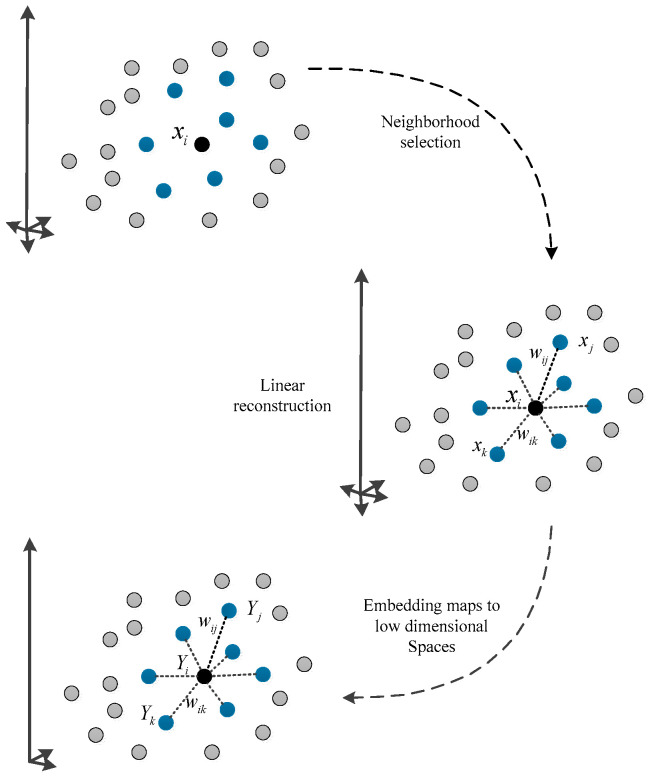
Illustrative description of local linear embedding algorithm.

LLE is a classical algorithm of manifold learning. It regards the linear reconstruction representation relationship between sample points and their local neighbors as the characterization of local geometric properties of manifolds. Its fundamental concept is to start with the local linearity of nonlinear data, based on which the global nonlinearity of the dataset is transformed into local linearity. According to the global structure information provided by the overlapping local neighborhoods, the global optimal low-dimensional embedding representation is sought while the local geometric relation of the data is fully preserved. The local linear embedding algorithm can learn the local structure of any dimension and is widely used in many applications such as fault detection and image recognition. Consequently, the application and improvement of the local linear embedding algorithm have been widely researched [6]. The LLE algorithm, as shown in Figure 1, includes the following three steps:

(1) Search for nearest neighbor points: The first step in the LLE algorithm is determining the nearest neighbors of each data point. Given a dataset $X=\left\{x_{1},x_{2},\ldots ,x_{N}\right\}\in {\mathbb{R}}^{D\times N}$, where D is the original dimensionality of the data and N is the number of samples. For each sample $x_{i}\in {\mathbb{R}}^{D\times 1}$, the neighbors are determined by either choosing the k closest Euclidean distance points or by selecting points within a fixed radius around the sample. These neighbors $N(x_{i})=\left\{x_{q_{i}^{1}},x_{q_{i}^{2}},\ldots ,x_{q_{i}^{k}}\right\}\in {\mathbb{R}}^{D\times k}$ are then used to form the neighborhood index set $Q\left(x_{i}\right)=\{q_{i}^{1},q_{i}^{2},\ldots ,q_{i}^{k}\}$ ensuring that the local relationships between data points are preserved.
(2) Calculate the optimal reconstruction weight matrix: each sample point is linearly reconstructed from its nearest neighbor data points $N\left(x_{i}\right)$, and the reconstruction error of all data points can be expressed in the following mathematical form:
  (1) $$\varepsilon (w)=\sum_{i=1}^{N}{\left\|x_{i}-\sum_{j\in Q\left(x_{i}\right)}w_{ij}x_{j}\right\|}^{2}$$
  where $w_{ij}$ is the weight between sample points $x_{i}$ and $x_{j}$, if $x_{j}\notin N(x_{i})$, $w_{ij}=0$, and the rows of the weight matrix $W\in {\mathbb{R}}^{N\times N}$ sum to one, that is $\sum_{j\in Q(x_{i})}w_{ij}=1$.
(3) Represent low-dimensional embedding information: The spatial position relationship between all sample points and the nearest neighbor set is kept unchanged in the mapping from high to low dimensions. Namely $w_{ij}$ is kept unchanged after linear reconstruction, and the low-dimensional reconstruction error is minimized.
  (2) $$\begin{array}{l} \min\phi (Y)=\sum_{i=1}^{N}{\left\|y_{i}-\sum_{j\in Q\left(x_{i}\right)}w_{ij}y_{j}\right\|}^{2},\\ s.t.\sum_{i=1}^{N}y_{i}=0\;and\;\frac{1}{N}YY^{T}=I_{d\times d} \end{array}$$
  where I is the identity matrix, $Y\in {\mathbb{R}}^{d\times N}$ is the embedding representation matrix of the sample point in the low-dimensional space, and $y_{i}\in {\mathbb{R}}^{d\times 1}$ is the *i*th column of the matrix Y, which represents the coordinate vector of the *i*th sample point in the low-dimensional space of dimension d. The constraint $\sum_{i=1}^{N}y_{i}=0$ is to ensure that the embedding coordinates are centered around the origin. This ensures that the low-dimensional embedding does not have a translation bias, and the data points are distributed around the origin in the embedding space. The constraint $\frac{1}{N}YY^{T}=I_{d\times d}$ ensures that the low-dimensional coordinates are scaled appropriately along each axis, and the variance of each axis is equal, preventing any one axis from disproportionately capturing the structure of the data. This is crucial for maintaining the intrinsic structure of the data after dimensionality reduction. The optimal solution Y of this object can be obtained by calculating the minimum d eigenvectors corresponding to the non-zero eigenvalues of the sparse, symmetric, semi-positive definite matrix $M={\left(I-W\right)}^{T}(I-W)$, where $W\in {\mathbb{R}}^{N\times N}$ is the optimal reconstruction weight matrix and ${\left(W\right)}_{ij}=w_{ij}$.

## 3. ALLRMM Model

LLE is a local processing method, which has the advantages of simplicity and high operational efficiency. In the process of sample dimension reduction, the neighborhood value is set as a fixed value for calculation. Nevertheless, the actual data are distributed on a heterogeneous manifold; the fixed neighborhood size is obviously unreasonable because it is only suitable for the homogeneous manifold structure. In addition, as a conventional manifold learning method, LLE neglects the modeling of data within and between manifolds and cannot deal with the problem of multi-manifold recognition of high-dimensional data. So considering the sensitive problem of neighborhood selection, this study takes a comprehensive view of the relationship between neighborhood size and sample density to further refine the criteria for distance selection. This approach considers the compactness and relative positional relationships of data points within the neighborhood, allowing for a more precise description of the local structure of the data points, thereby making neighborhood selection more accurate and reasonable. To solve the problem that multi-manifolds cannot be processed, the within-manifold graph and between-manifold graph are constructed by combining the data labels, based on which the spacing of multi-manifolds is measured. By maximizing the spacing of manifolds and minimizing the within-class differences, the feature dimension reduction extraction of high-dimensional feature datasets is realized. The improvement of LLE mainly includes the following two aspects:

### 3.1. Optimization of Local Neighborhood Selection Method

The first step of the manifold learning algorithm is domain selection, and appropriate domain selection is key to the algorithm’s success. The algorithm assumes that each data point and its nearest neighbor are in the same linear hyperplane. Accordingly, it takes the linear reconstruction representation relationship between the sample point and its local nearest neighbor as the description of the local geometric property of the manifold. Fundamentally, the algorithm starts with the local linearity of nonlinear data and converts the global nonlinearity of the dataset into local linearity. Global structure information is provided according to overlapping local fields, and a global optimal low-dimensional embedding representation is sought while the local geometric relation of data is fully maintained [21]. Furthermore, a density scaling factor algorithm is proposed to solve the sensitive problem of neighborhood selection in conventional manifold learning. The scaling factor is used to adjust any initial domain value to the ideal neighborhood value of each sample point according to the neighborhood iteration factor, taking full account of the relationship between high-dimensional data density distribution and neighborhood value.

**Figure 2:**
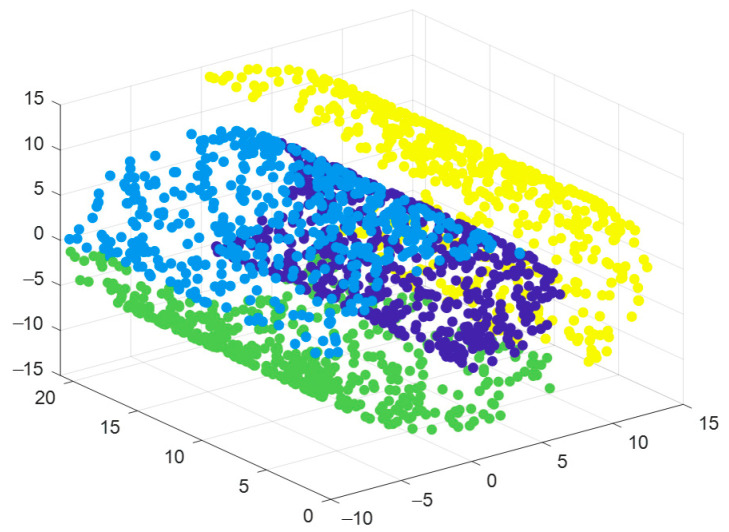
Swiss Roll dataset.

**Figure 3:**
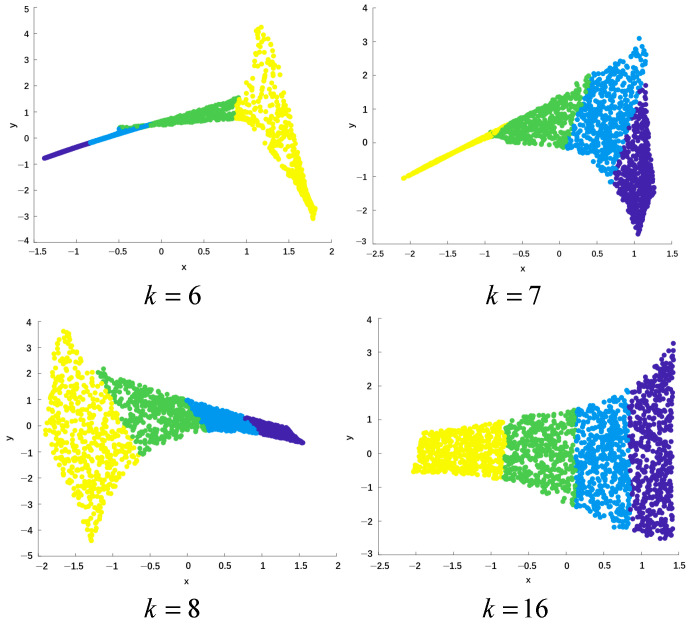
Sensitivity of LLE algorithm to the neighborhood parameters k.

(1) **Sensitivity analysis of neighborhood selection:** Generally, an optimal domain size should be taken. If the domain size is too small, the continuous topological space will be constructed into multiple separated subgraphs, resulting in the loss of connections between certain points and the failure to reflect the global characteristics. If it is too large, the neighborhood of certain sample data points will come from other folding planes.
  Next, the influence of the size of the neighborhood parameter k on data dimensionality reduction will be elaborated in detail through Figure 2 and Figure 3.

Figure 2 shows the classic Swiss Roll dataset, a two-dimensional manifold dataset often used in manifold learning research. Its representation in three-dimensional space resembles a rolled-up paper scroll. Figure 2 is a three-dimensional scatter plot, with the X-axis, Y-axis, and Z-axis representing the coordinates in three-dimensional space. The figure uses color coding to display the distribution of data points in three-dimensional space, where colors represent different labels, helping us intuitively understand the global structure and local features of the data.

Figure 3 further explores the sensitivity of the Local Linear Embedding (LLE) algorithm to the neighborhood parameter k. LLE is a popular manifold learning method that aims to achieve low-dimensional embedding of high-dimensional data by preserving the local geometric relationships between data points. Figure 3 shows the results of the LLE algorithm reducing the Swiss Roll dataset from three dimensions to two dimensions under different k values. Each subgraph corresponds to a specific k value, and the X-axis and Y-axis represent the coordinates in the two-dimensional space. By checking the dimensionality reduction results under different k values, one can observe the significant impact of the k value selection on the performance of the LLE algorithm. If k is too small, the linearity of the nearest neighbor region is guaranteed, but the global property of the data cannot be reflected. If k is too large, the premise that the algorithm can learn local linearity of the manifold does not hold. Therefore, it can be seen that the selection of the nearest neighbor number significantly affects the dimensionality reduction effect of the LLE algorithm.

Thus, the linear processing method is adopted for most of the nonlinear distributed data, resulting in damage to the nonlinear structure between the original data.

**Figure 4:**
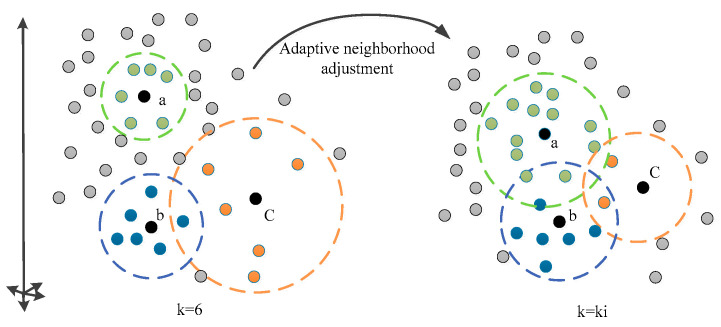
Effect of sample density on neighborhood size.

(2) **Sample density analysis:** As shown in Figure 4, owing to the unevenly distributed manifold structure, overlapped neighborhoods are conducive to information transmission and the protection of the local linearity hypothesis. Therefore, to maximize overlap, four places with high local density of sample points and more sample points in the neighborhood are selected to make the neighborhood larger, resulting in neighborhood overlap.

For places with low local density of sample points, a few neighborhood sample points are picked to make the neighborhood smaller and prevent too many local neighborhoods from destroying the local linearity hypothesis of manifold learning. In Figure 4, assuming that the initial fixed value is 6, it can be found that the local density of sample points a and b is large, while the sample density of point c is small. Selecting a fixed field parameter value of 6 will make the neighborhood of points a and b with high local density too small, and the manifold will be divided into multiple discontinuous subgraphs. The neighborhood of point c with small local density is too large, which destroys the assumption of local linearity. So after adaptive neighborhood iteration, the value k changed from the original 6 to $k_{i}$, the neighborhood number of points a and b is increased, and that of point c is reduced, so that different neighborhoods overlap on the premise of maintaining local linearity, and information is transmitted between neighborhoods.

(3) **Adaptive neighborhood selection:** The density scaling factor of each sample point was calculated according to the density scaling factor algorithm proposed in the previous section, and the initial neighborhood value was adjusted adaptively according to the size of the density scaling factor. Consequently, the neighborhood value parameter of the high-density sample point increased while that of the low-density sample point decreased. Considering that the extreme value of the initial neighborhood value significantly affects the adaptive results, the initial extreme neighborhood value is preliminarily adjusted, followed by the adaptive iteration to get the ideal neighborhood value. The initial neighborhood value k is based on the features of the datasets. The *k* value adaptation is performed using the density scaling factor algorithm. The density scaling factor $\alpha_{i}$ is computed based on the local density of sample points in the high-dimensional space. As the local density of a sample point increases, $\alpha_{i}$ becomes larger; conversely, as the inter-sample density decreases, $\alpha_{i}$ becomes smaller. The adaptive iteration process is determined as follows:

According to the distance between the sample points, calculate the local density of each sample point, and the local density calculation formula:

(3) $$\rho_{i}=\sum_{j}\eta (d_{ij}-d_{c})$$

where $d_{ij}$ is the set cut-off distance. If $d_{ij}-d_{c}\leq 0,\;\eta (\cdot )=1$, if $d_{ij}-d_{c}>0,\;\eta (\cdot )=0$. That is, the number of data points less than the cut-off distance $d_{c}$ is used to represent the local density $\rho_{i}$.

The density scaling factor $\alpha_{i}$ depends on the local density of the sample point i, and the calculation formula is as follows:

(4) $$\alpha_{i}=\exp(\frac{\rho_{i}-\bar{\rho }}{s})$$

(5) $$\bar{\rho }=\frac{1}{n}\sum_{i=1}^{n}\rho_{i}$$

(6) $$s=\frac{1}{n-1}\sum_{i=1}^{n}{\left(\rho_{i}-\bar{\rho }\right)}^{2}$$

In the formula, $\bar{\rho }$ is the average density of the sample points, and s is the sample variance.

Using a formula, we calculate a value β to determine whether the initial neighborhood value k is an extreme value. β is calculated as

(7) $$\beta =\exp(\frac{\bar{\rho }-k}{k})$$

If $\beta >>{\bar{\alpha }}_{i}$, the initial neighborhood value k is too small, and the value of k needs to be increased:

(8) $$k_{i(j-1)}=k_{i(j-2)}*2$$

If $\beta <<{\bar{\alpha }}_{i}$, the initial neighborhood value k is too large, and the value of k needs to be reduced:

(9) $$k_{i(j-1)}=k_{i(j-2)}/2$$

where $k_{i(j-1)}$ represents the (*j* − 1)th initial iteration of the initial neighborhood value k of the sample point $x_{i}$, and ${\bar{\alpha }}_{i}$ is the scaling factor for the average density.

Subsequently, the adjusted neighborhood value β is calculated. If the iteration condition $\left|\beta -{\bar{\alpha }}_{i}\right|\leq \delta$ is not met, the extreme value is adjusted until the adjusted neighborhood value is no longer the extreme value, following which iterations are made based on the density scaling factor. The neighborhood value k of each sample point can be adjusted adaptively as follows:

(10) $$\begin{array}{l} k_{ij}=k_{i(j-1)}*a_{i},i=1,2,\ldots ,n\\ s.t.\left|\beta -{\bar{\alpha }}_{i}\right|\leq \delta \end{array}$$

where $k_{ij}$ represents the *j*th adaptive iteration of the initial neighborhood value k of the sample point $x_{i}$; δ is the limiting condition for judging whether the adaptive adjusted neighborhood value is ideal.

**Figure 5:**
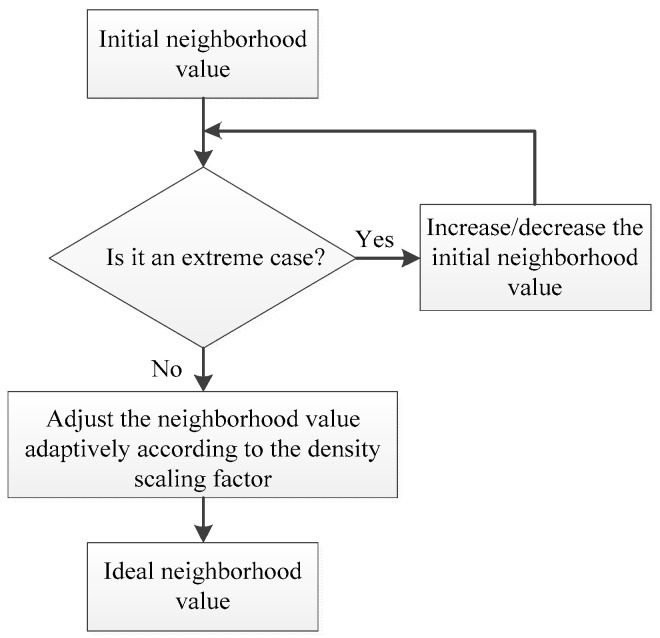
Adaptive neighborhood selection flowchart.

The adaptive neighborhood value iteration algorithm adapts the globally consistent initial neighborhood value to obtain the ideal neighborhood value of each sample point. According to the above calculation and adaptive iteration process based on the density scaling factor, the flow chart of the adaptive neighborhood iteration algorithm is shown in Figure 5.

The steps are as follows:

Step 1: Calculate the local density and density scaling factor of each sample point according to the formula.

Step 2: Determine whether the initial neighborhood value is an extreme value using (7); if it is an extreme value, adjust the neighborhood value using (8) or (9) and proceed to step 3; if it is not an extreme value, directly proceed to step 3.

Step 3: Adaptively adjust the neighborhood value size of each sample point $x_{i}$ according to the density scaling factor of each sample point X according to (10), ensuring that the neighborhood value parameter of the high-density sample point increases and that of the low-density sample point decreases. Consequently, the ideal neighborhood value $k_{i}$ is obtained.

### 3.2. Improved Edge Margin Algorithm for Local Linear Embedded Manifolds

The conventional manifold learning method neglects the modeling of data within and between manifolds and fails to deal with the problem of multi-manifold recognition of high-dimensional data. Therefore, this paper proposes an improved Locally Linear Representation of Manifold Margins (LLRMM) [22,23] algorithm that constructs within-manifold and between-manifold graphs based on data labels, measuring the spacing of multi-manifolds on this basis. The algorithm achieves feature dimensionality reduction extraction for high-dimensional feature datasets by maximizing the spacing of manifolds and minimizing within-class differences. The core of the algorithm is built on LLE ALLRMM first fits the local linear model and then aligns the global coordinates. The global nonlinear manifold is divided into some small local linear sub-blocks, and then these sub-blocks are globally arranged together through information transfer to obtain a consistent low-dimensional manifold representation of the data. Compared to other algorithms, the proposed algorithm can adaptively divide into different sizes of sub-blocks, offering greater flexibility and adaptability while better accounting for the inhomogeneity of real data. As a result, it performs exceptionally well when handling rotating machinery fault datasets.

(1) **The construction of manifold graphs:** The within-manifold graphs reflect the local relations in the manifold comprising similar data. For any sample point $X_{i}$ (the *i*-th column of dataset X) in the within-manifold graph, sample points with the same category and minimum distance $k_{i}$ are selected to form the local neighborhood of sample point $X_{i}$. Therefore, in this neighborhood, similar to that in LLE, the minimum linear error is expressed by the closest neighbor points of the same class of sample point $X_{i}$.
  (11) $$\varepsilon \left({\left(W_{w}\right)}_{i}\right)=\min{\left\|X_{i}-\sum_{j\in Q\left(X_{i}\right)}{\left(W_{w}\right)}_{ij}X_{j}\right\|}^{2},s.t.\sum_{j\in Q\left(X_{i}\right)}{\left(W_{w}\right)}_{ij}=1$$
  where $X_{j}$ is the nearest neighbor of the sample point $X_{i}$ in the within-manifold graph; $k_{i}$ is the adaptive neighborhood selection result of sample point $X_{i}$; ${\left(W_{w}\right)}_{i}=\left(w_{i1},w_{i2},\ldots ,w_{iN}\right)\in {\mathbb{R}}^{1\times N}$ is the minimum linear representation weight vector of the same kind as the nearest neighbor of sample point $X_{i}$.

Since the local weights ${\left(W_{w}\right)}_{ij}$ sum to one, the formula can be rewritten as follows:

(12) $$\begin{array}{c} \varepsilon \left({\left(W_{w}\right)}_{i}\right)=\min{\left\|\sum_{j\in Q\left(X_{i}\right)}{\left(W_{w}\right)}_{ij}X_{i}-\sum_{j\in Q\left(X_{i}\right)}{\left(W_{w}\right)}_{ij}X_{j}\right\|}^{2}\\ =\min{\left\|\sum_{j\in Q\left(X_{i}\right)}{\left(W_{w}\right)}_{ij}\left(X_{i}-X_{j}\right)\right\|}^{2}\\ \;=\min\left\{\sum_{j\in Q\left(X_{i}\right)}{\left(W_{w}\right)}_{ij}\left(X_{i}-X_{j}\right)\cdot \sum_{t\in Q\left(X_{i}\right)}{\left(W_{w}\right)}_{it}\left(X_{i}-X_{t}\right)\right\}\\ \;=\min\sum_{j\in Q\left(X_{i}\right)}\sum_{t\in Q\left(X_{i}\right)}{\left(W_{w}\right)}_{ij}{\left(W_{w}\right)}_{it}G_{jt} \end{array}$$

where $G_{jt}={\left(X_{i}-X_{j}\right)}^{T}\left(X_{i}-X_{t}\right)$ represents the local term of the Gram matrix $G\in {\mathbb{R}}^{k_{i}\times k_{i}}$, which can be expressed as

$${\left(\begin{array}{ccc} G_{11} & \ldots & G_{1k_{i}}\\ \vdots & \ddots & \vdots \\ G_{k_{i}1} & \ldots & G_{k_{i}k_{i}} \end{array}\right)}_{k_{i}\times k_{i}}$$

Using the Lagrange multiplier method, the locally linear representation weights in the within-manifold graph can be obtained.

Firstly, a Lagrange function can be formed as

(13) $$L\left({\left(W_{w}\right)}_{i}\right)=\varepsilon \left({\left(W_{w}\right)}_{i}\right)-\lambda \left(\sum_{j\in Q\left(X_{i}\right)}{\left(W_{w}\right)}_{ij}-1\right)$$

Then let $\frac{\partial L}{\partial {\left(W_{w}\right)}_{i}}=0$, the locally linear representation weights in the within-manifold graph can be deduced to the following formulation.

(14) $${\left(W_{w}\right)}_{ij}=\left\{\begin{array}{l} \frac{\sum_{t\in Q\left(X_{i}\right)}G_{jt}^{-1}}{\sum_{j\in Q\left(X_{i}\right)}\sum_{t\in Q\left(X_{i}\right)}G_{lm}^{-1}},X_{j}\in WithinM(X_{i})\\ 0,\;otherwise \end{array}\right.$$

where $WithinM(X_{i})$ represents the within-manifold graph neighborhood of the manifold of sample point $X_{i}$; $G_{jt}^{-1},G_{lm}^{-1}$ represents the local term of the inverse Gram matrix $G^{-1}\in {\mathbb{R}}^{k_{i}\times k_{i}}$.

In the within-manifold graph, the minimum linear representation weight matrix $W_{w}$ is obtained by repeating the same steps for each point. The within-manifold and between-manifold graphs are constructed in a similar manner; the process is repeated except that the neighborhood points should be selected from different manifolds. The weight formula of the local minimum linear representation is shown in (12) and (14). The minimum linear representation weight matrix $W_{b}$ of a between-manifold graph can also be obtained.

(15) $${\left(W_{b}\right)}_{ij}=\left\{\begin{array}{l} \frac{\sum_{t\in Q\left(X_{i}\right)}G_{jt}^{-1}}{\sum_{j\in Q\left(X_{i}\right)}\sum_{t\in Q\left(X_{i}\right)}G_{lm}^{-1}},X_{j}\in BetweenM(X_{i})\\ 0,\;otherwise \end{array}\right.$$

where $BetweenM(X_{i})$ represents the between-manifold graph neighborhood of sample point $X_{i}$; $W_{b}$ is the weight matrix in the between-manifold graph.

In summary, the within-manifold and between-manifold graphs can be constructed by corresponding minimum linear representation weights between point pairs. In a manifold diagram, any sample can be linearly represented by the nearest neighbor points of its within-manifold graph, and the manifold divergence matrix represents the degree of aggregation between data. Therefore, the minimum linear representation error of the manifold graph can be used to represent the manifold divergence matrix of the within-manifold graph, which can be defined as shown below:

(16) $$S_{w}=XU_{w}X^{T}$$

(17) $$S_{b}=XU_{b}X^{T}$$

where $U_{w}={\left(I-W_{w}\right)}^{T}(I-W_{w})$ $U_{b}={\left(I-W_{b}\right)}^{T}(I-W_{b})$; $W_{w}$ and $W_{b}$ represent the minimum representation error weight matrices of the within-manifold and the between-manifold graphs, respectively; $S_{w},S_{b}$ represents the graph divergence matrix within and between manifolds, respectively; and $X\in {\mathbb{R}}^{D\times N}$ represents the original high-dimensional data matrix and $U_{w},U_{b}\in {\mathbb{R}}^{N\times N}$.

(2) **Manifold margin definition:** To describe the degree of dispersion between manifolds with different class labels, a new manifold margin is defined. The manifold margin is the distance between points on the manifold $M_{i}$ and other manifolds M minus the within-manifold graph distance of the manifold $M_{i}$. The within-manifold graph distance of the manifold can be described as the degree of convergence and dispersion inside the manifold and can be expressed by the within-manifold graph divergence matrix of the manifold. Therefore, the definition of manifold margin is as shown below:
  (18) $$S_{M}=\sum_{i,j}d(M_{i},M_{j})-\sum_{i}tr\left(M_{i}\right)$$
  where $S_{M}$ stands for manifold margin; $d(M_{i},M_{j})$ represents the distance between manifold $M_{i}$ and manifold $M_{j}$; $tr\left(M_{i}\right)$ is taken to represent the scale of manifold $M_{i}$; and $d(M_{i},M_{j})$ is defined as the sum of the minimum distance between every point on the ith manifold $M_{i}$ to other manifolds $M_{j}\left(j=1,2,\ldots ,c,i\neq j\right)$:
  (19) $$d(M_{i},M_{j})=\sum_{i}\left\{\min d(X_{i},M_{j})\right\}$$
  where $X_{i}$ is any sample point on the *i*th manifold $M_{i}$.

The minimum distance between the sample point $X_{i}$ and other manifolds can be expressed by the square of the weighted average of any sample point $X_{i}$ and its nearest neighbors of the between-manifold graph. On repeating this process for all sample points, the following formula for $d(M_{i},M_{j})$ is obtained.

(20) $$d(M_{i},M_{j})={\sum_{i}\left\|X_{i}-\sum_{j\in Q\left(X_{j}\right)}{\left(W_{b}\right)}_{ij}X_{j}\right\|}^{2}$$

The above formula is actually the divergence matrix of the graph between manifolds. Thus, the spacing between manifolds can be expressed as

(21) $$S_{M}=S_{b}-\sum_{i}tr\left(M_{i}\right)$$

Furthermore, $\sum_{i}tr\left(M_{i}\right)=\sum_{i}\varepsilon \left({\left(W_{w}\right)}_{i}\right)=tr\left(XU_{w}X^{T}\right)$, so $S_{M}$ can be written as follows:

(22) $$S_{M}=S_{b}-S_{w}=X(U_{b}-U_{w})X^{T}$$

(3) **Optimization solution:** According to the within-manifold and between-manifold graph divergence matrices, and the previously defined manifold edge distance, the purpose of the proposed algorithm is to find a subspace on which data of different manifolds can be more easily distinguished. That is, the edge distance of manifolds should be maximized in the low-dimensional subspace, and the within-class difference should be minimized.

Therefore, to solve the low-dimensional subspaces, the above two objective functions must be satisfied, which gives the following formula:

(23) $$\left\{\begin{array}{c} \max tr\{Y(U_{b}-U_{w})Y^{T}\}\\ \min tr\{YU_{w}Y^{T}\} \end{array}\right.$$

where $tr\left(\cdot \right)$ denotes the trace of a matrix, which is the sum of its diagonal elements.

Subsequently, a single-objective optimization problem was obtained, whose form is shown in the following formula:

(24) $$\max\frac{tr\{Y(U_{b}-U_{w})Y^{T}\}}{tr\{YU_{w}Y^{T}\}}$$

Generally, the conventional manifold learning method encounters out-of-sample problems. Thus, it refers to linear change, A, between the original data and the embedded data, such as $Y=A^{T}X$. This linear transformation is orthogonally restricted, that is $AA^{T}=I$. Thus, (20) is rewritten as

(25) $$\left\{\begin{array}{l} \max\frac{tr\{A^{T}X(U_{b}-U_{w})X^{T}A\}}{tr\{A^{T}XU_{w}X^{T}A\}}=\max\frac{tr\{A^{T}S_{M}A\}}{tr(A^{T}S_{w}A)}\\ s.t.\;A^{T}A=I \end{array}\right.$$

where A is the linear transformation matrix. The objective of this optimization is to find a low-dimensional subspace that maximizes the manifold edge distance between different categories of data Y after dimensionality reduction and minimizes the global manifold error. The Lagrange multiplier method is used to solve the above constraint function, and the feature decomposition (22) is obtained. A comprises the feature vectors corresponding to the d largest eigenvalues before the feature decomposition.

(26) $$S_{M}A_{i}=\lambda_{i}S_{w}A_{i}$$

where $\lambda_{i}$ is the eigenvalue of the generalized characteristic equation, $S_{M}$ is the manifold margin, and $A_{i}$ is a characteristic vector.

Finally, the characteristic data after dimensionality reduction can be obtained as follows.

(27) $$Y=A^{T}X$$

**Figure 6:**
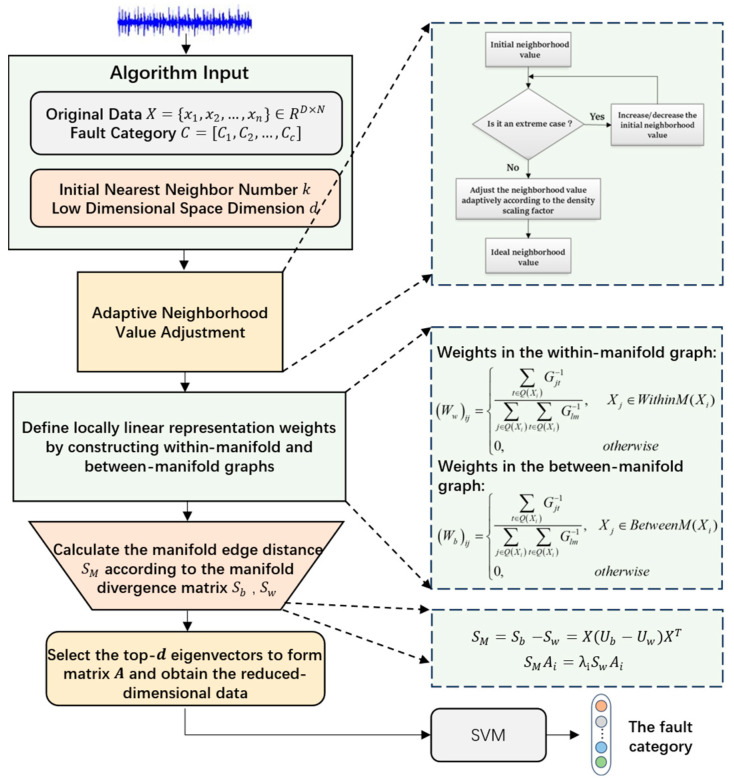
The overall flowchart of the ALLRMM algorithm.

Therefore, considering the contribution to the classification effect of the algorithm, a subspace will be sought to make it easier to distinguish the data of different manifolds on this subspace, maximize the distance between different manifolds, and minimize the within-class distance within manifolds. That is, the edge distance of manifolds is maximized, and the divergence matrix of graphs within manifolds is minimized in the low-dimensional subspace. Figure 6 is the overall flowchart of the ALLRMM algorithm.

The steps to improve the algorithm (Algorithm 1) are as follows:
**Algorithm 1** Algorithm for ALLRMM **Input:** original data $X=\{x_{1},x_{2},\ldots ,x_{N}\}\in {\mathbb{R}}^{D\times N}$, data category $C=[C_{1},C_{2},\ldots ,C_{c}]$, initial nearest neighbor number k, low-dimensional space dimension d; **Output:** Transform matrix A and data Y after dimensionality reduction. *Step 1:* Determine the domain size of each data sample point $k_{i}$ adaptively through density scaling factor $\alpha_{i}$; *Step 2:* Construct the within-manifold and between-manifold graphs by adaptive selection of k. *Step 3:* Calculate the weight matrix $W_{b},W_{w}$ of the corresponding between-manifold and within-manifold graphs using the formula and calculate the corresponding divergence matrix $S_{b},S_{w}$; *Step 4:* Calculate the manifold edge distance $S_{M}$ according to the manifold divergence matrix; *Step 5:* Solve the feature decomposition equation $S_{M}A_{i}=\lambda_{i}S_{w}A_{i}$. The eigenvectors corresponding to the first d maximum eigenvalues are selected to form the transformation matrix A and obtain the data after dimensionality reduction by solving $Y=A^{T}X$.

### 3.3. Analysis of the Initial Dimensionality Reduction Effect of Multi-Manifolds

The Swiss roll data can be used to evaluate the effectiveness of dimensionality reduction algorithms, as its locally Euclidean manifold structure enables meaningful computation of Euclidean distances between neighboring points. However, the existing nonlinear manifold dimensionality reduction methods can deal with nonlinear structure data well to a certain extent, but they are limited to well-separated structures. How to deal with the general situation where the data contains linear and nonlinear structures and overlaps with each other in the real situation, this paper constructs an overlapping dataset of Swiss Roll data and Sigmoid data for practice. Under the condition of an ideal initial neighborhood value, the dimensionality reduction effects of multi-manifold, KPCA, LLE, and ALLRMM were compared.

**Figure 7:**
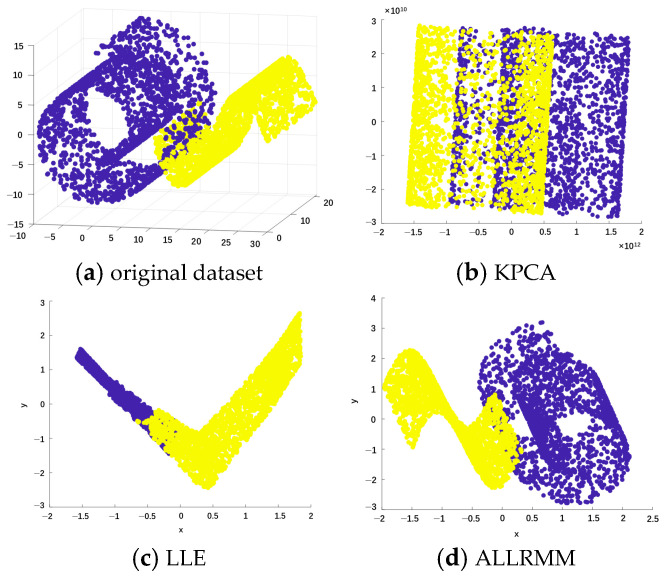
Mixed manifold structure dataset and rendering effect of dimensional reduction.

Figure 7 shows the mixed manifold structure dataset and the effects after being processed by different dimensionality reduction methods. The original dataset needs to be better presented in a three-dimensional space to show the separation and distribution of the data. Two-dimensional graphs are used to display the results after dimensionality reduction, as most dimensionality reduction methods aim to map high-dimensional data to a two-dimensional space for visualization and analysis. Through these graphs, the effects of different dimensionality reduction methods in handling the mixed manifold structure dataset can be intuitively compared, especially their ability to distinguish different manifolds or categories. Figure 7a presents the original dataset of the mixed manifold structure; different colors in the dataset represent different manifolds. The X-axis, Y-axis, and Z-axis, respectively, represent the three original features of the data, which are the natural attributes of the data generation process and are used to describe the position of the samples in the three-dimensional space. This figure is used to show the distribution of the data before any dimensionality reduction processing. The following three subfigures, respectively, show the results after using KPCA, LLE, and the ALLRMM method proposed in this paper. The coordinate axes represent the two main components after dimensionality reduction. The figure shows the distribution of the data points after dimensionality reduction, and different colors represent different manifolds.

The results show that on the multi-manifold dataset, the original topology structure is damaged after the dimensionality reduction using KPCA, and multi-manifolds cannot be recognized. Similarly, after dimension reduction using the conventional manifold learning LLE algorithm, multi-manifolds cannot be recognized. In contrast, the ALLRMM algorithm preserves the structure of the multi-manifold.

### 3.4. Complexity

The computational complexity of the ALLRMM is composed of five parts. Let the sample size be n, the original feature dimension be d, the maximum neighborhood number be K (K≪n), and $O(n^{2}\cdot d+n\cdot K^{3}+d^{3})$ be the total complexity. The first step is to calculate the local density: Firstly, a distance matrix of size n×n needs to be calculated. For each pair of distances, d floating-point operations are required, so the time complexity is $O(n^{2}\cdot d)$. Next is adaptive adjusting neighborhood: Scan each sample’s n distance records and update K. The overall complexity remains $O(n^{2})$. The third step of constructing intra-class and inter-class graphs: After extracting K neighbors for each of the n samples, solve the K×K linear equation system to obtain the reconstructed weights. The single-time complexity is $O(K^{3})$, and the total complexity is $O(n\cdot K^{3})$. The fourth step of generating a d×d divergence matrix: Each sample participates in the external accumulation addition, resulting in $O(n\cdot d^{2})$ operations. The last step of generalized eigenvalue decomposition: perform eigenvalue calculation on the d×d matrix. The standard algorithm complexity is $O(d^{3})$.

Compared to traditional manifold learning methods such as LLE and KPCA, ALLRMM requires additional storage for the between-manifold edge distances and the disparity matrices between manifolds, resulting in higher spatial demands. However, these additional storage requirements significantly enhance the representational capacity of the data structure, enabling the algorithm to better capture the differences between manifolds and the intrinsic structure of the data. Although the ALLRMM’s spatial complexity increases when handling large-scale data, adaptive neighborhood selection and optimization of between-manifold distances effectively reduce the storage of redundant data, thereby improving spatial utilization efficiency.

## 4. Experimental Design

### 4.1. Dataset

In order to verify the applicability of the fault diagnosis method based on multi-manifold learning and its diagnostic performance under variable working conditions, the bearing fault data of Professor Lei Yaguo’s team from Xi’an Jiaotong University and the Ottawa variable working condition dataset were selected for experiments. The test bearing for the XJTU-SY dataset was LDK UER204. A total of 15 bearings were designed under 3 working conditions, and the speed of the selected working conditions was 2400 (r/min). Bearing life data under a 10 kN load, sampling frequency is 25.6 kHz, sampling interval is 1min, sampling time is 1.28 s, and the number of samples each time is 32,768. The fault data includes seven states: normal, early inner ring fault, moderate inner ring fault, severe inner ring fault, early outer ring fault, moderate outer ring fault, and severe outer ring fault. The Ottawa dataset is shown in Table 1. The dataset is designed under four variable conditions: increasing speed, decreasing speed, increasing then decreasing speed, and decreasing then increasing speed. The data sampling frequency is 20 kHz, sampling time is 10s, and the data includes bearing normal, inner ring failure, and outer ring failure, three states. Variable working conditions 1 and 4 were selected for experiments. According to the 1024 data points of each sample, samples in different states were divided into 30 samples, and 20 samples were randomly selected as the training set and 10 samples as the test set. The 30-dimensional features shown in Table 2, Table 3 and Table 4 are also selected.

### 4.2. Data Preprocessing

Per sample has 1024 vibration signal data points; samples from different states were divided into 30 segments, with 20 randomly selected as the training set and 10 as the test set. To enhance fault features and suppress noise, the segments were processed using Minimum Entropy Deconvolution (MED) filtering. The 30-dimensional features shown in Table 2, Table 3 and Table 4 are also selected. Table 2 presents 16 statistical features extracted from the time domain, including mean and peak value, which reflect the amplitude distribution, waveform symmetry, and variation trends of the vibration signals. Table 3 describes five frequency-domain features, such as the frequency center, to characterize the spectral energy distribution. Prior to computing frequency-domain features, each signal segment undergoes a Fast Fourier Transform (FFT) to determine its frequency spectrum. Table 4 provides nine multifractal features that capture signal complexity and multi-scale variation through the singularity spectrum and Holder exponents. These features, derived from the MED-filtered signal segments, serve as the foundation for subsequent feature selection and classification modeling.

## 5. Analysis of Experimental Result

### 5.1. Analysis of the Dimension Reduction Effect of Multi-Manifold

First, the density proportion factor algorithm is employed to determine the optimal neighborhood parameter values k.

**Figure 8:**
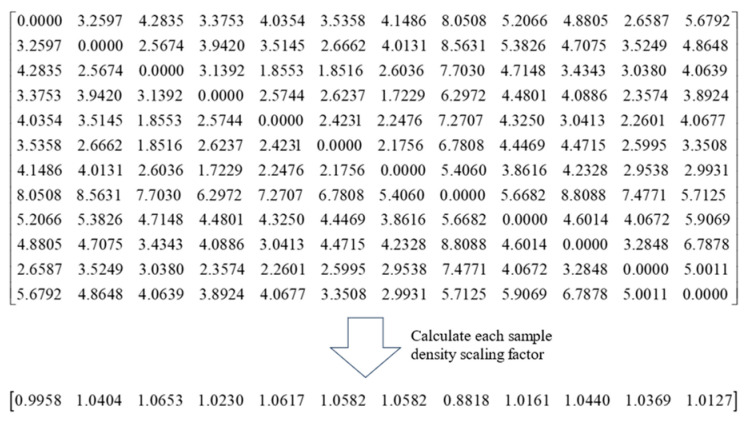
Sample point neighborhood parameter selection.

In Figure 8, it is the distance matrix of sample points and the density scaling factor $\alpha_{i}$ calculated according to (3)–(6). Each element represents the Euclidean distance between sample points $x_{i}$ and $x_{j}$.

To simplify processes and ensure robustness, set the truncation distance dc=8, the neighborhood restriction condition δ to 2, and calculate the density scaling factor of all sample points as shown. The initial neighborhood value is 12, taking sample point $x_{1}$ as an example, β=0.6695, and the average density scaling factor ${\bar{\alpha }}_{i}=3.3749$. The condition $\beta -{\bar{\alpha }}_{i}\leq -\delta$ is judged by the extreme value of the initial neighborhood value. So adjust the initial neighborhood value to 6 according to (9), repeat the above steps, and finally adjust the initial neighborhood value to 3. Repeat the above steps for each sample point to obtain the ideal neighborhood value of each sample point.

**Figure 9:**
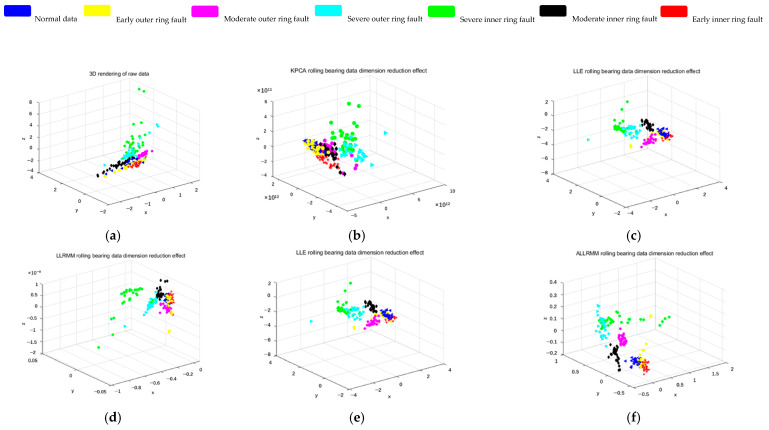
XJTU-SY: (**a**) T-SNE dimension reduction effect of the original data. (**b**) KPCA dimension reduction effect. (**c**) LLE dimension reduction effect. (**d**) LLRMM dimension reduction effect. (**e**) UMAP dimension reduction effect. (**f**) ALLRMM dimension reduction effect.

**Figure 10:**
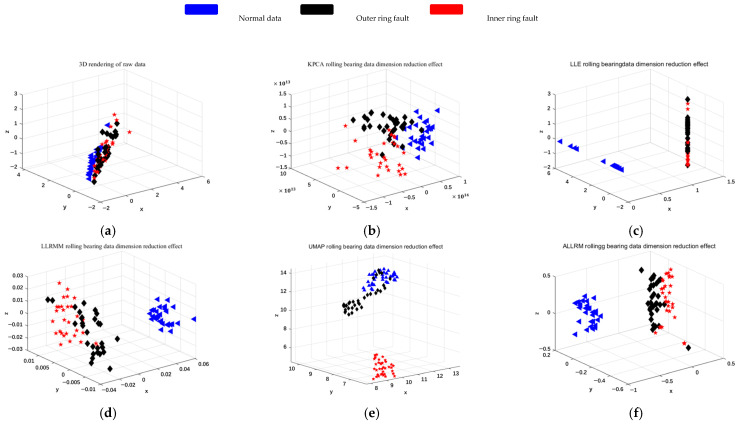
Ottawa-1: (**a**) T-SNE dimension reduction effect of the original data. (**b**) KPCA dimension reduction effect. (**c**) LLE dimension reduction effect. (**d**) LLRMM dimension reduction effect. (**e**) UMAP dimension reduction effect. (**f**) ALLRMM dimension reduction effect.

**Figure 11:**
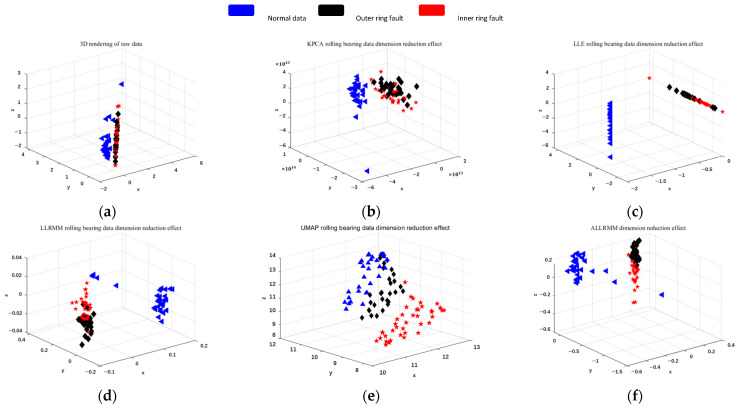
Ottawa-4: (**a**) T-SNE dimension reduction effect of the original data. (**b**) KPCA dimension reduction effect. (**c**) LLE dimension reduction effect. (**d**) LLRMM dimension reduction effect. (**e**) UMAP dimension reduction effect. (**f**) ALLRMM dimension reduction effect.

As shown in Figure 9, Figure 10 and Figure 11, three different datasets are selected, and the classical dimensionality reduction algorithms T-SNE, KPCA, LLE, LLRMM, and UMAP are used to compare with the algorithm in this paper. The data is normalized, and the coordinates are inconsistent because KPCA is mapped by a kernel function.

In order to prove the effectiveness of the method, the XJTU-SY bearing fault dataset was selected. Figure 9 shows the XJTU-SY dataset, and six types of fault data are selected for experiments. Blue represents normal data, red represents an early inner ring fault, and black represents a moderate inner ring fault. Green indicates a severe fault in the inner ring, yellow indicates an early fault in the outer ring, purple-red indicates a moderate fault in the outer ring, and light blue indicates a severe fault in the outer ring. It can be clearly seen from Figure 9 that the proposed ALLRMM algorithm distinguishes the fault data obviously and has less overlap. As shown in Figure 9a–c, for the T-SNE, KPCA, and LLE algorithms, the blue, red, and yellow patterns have obvious overlap, which makes it difficult to distinguish early faults. As shown in Figure 9e–f, the blue, red, and yellow patterns have less overlap, which is more obvious than that in Figure 9b–d, and Figure 9f is more obvious than that in Figure 9e in distinguishing moderate and severe faults.

Figure 10 shows experimental results under a single working condition. In order to verify the applicability of the proposed algorithm, for variable working condition data, the Ottawa variable working condition bearing dataset is selected to conduct experiments with different dimensionality reduction algorithms.

The results are shown in Figure 11, where blue represents normal data, red and black represent bearing inner ring failure and outer ring failure faults, respectively. It can be seen from the figures that after dimension reduction, the multi-manifold learning dimensionality reduction algorithm has a better effect on distinguishing faults than the traditional KPCA and LLE.

Observing Figure 10a–c and Figure 11a–c of the two figures, it can be found that under the re-varying operating conditions, the patterns of the traditional manifold learning algorithms T-SNE, KPCA, and LLE overlap obviously, which cannot effectively distinguish different faults. By comparing Figure 10d–f and Figure 11d–f, it can be found that ALLRMM calculated by multi-manifold learning can effectively distinguish normal data from fault data, but there is obvious overlap between different fault data. By improving the optimization formula and neighborhood selection, different faults can be distinguished significantly.

In summary, after three kinds of actual data verification, the superiority of the proposed algorithm is proved.

### 5.2. Analysis of Algorithm Recognition Accuracy

**Figure 12:**
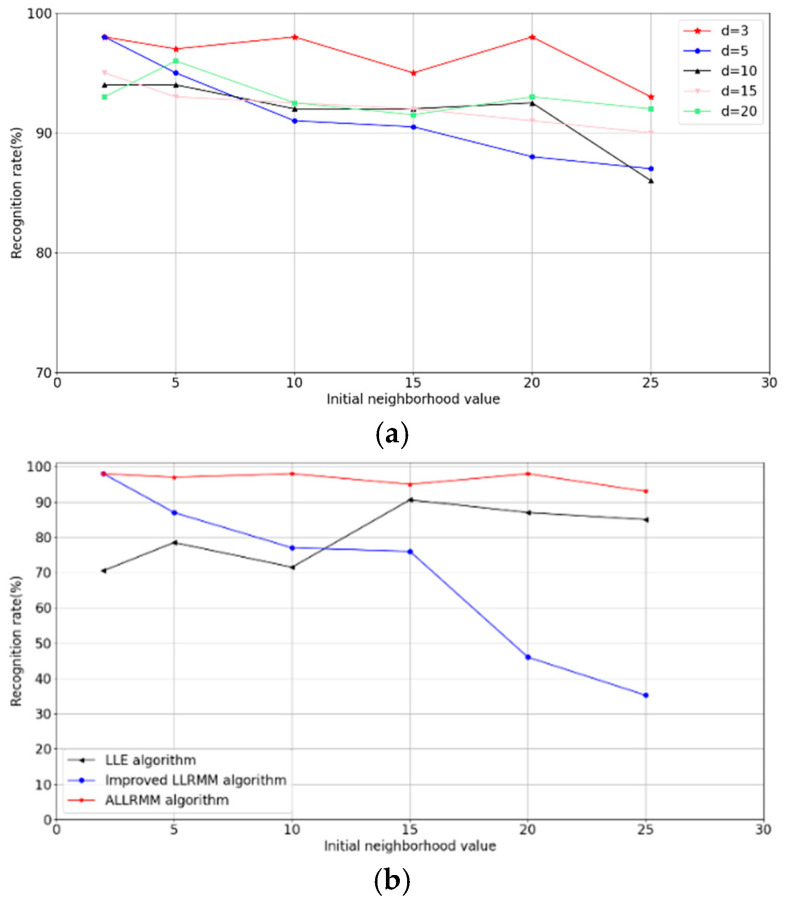
(**a**) The recognition rate of ALLRMM algorithm on different initial neighborhood values. (**b**) The recognition rate of three algorithms on rolling bearing fault datasets with different initial neighborhood values.

To further compare the dimensionality reduction effect of these four algorithms, Support Vector Machine (SVM) is employed to perform pattern recognition of the features extracted by the above algorithms, and its recognition performance is shown according to the experiment. In this experiment, two-thirds of the samples of rolling bearing data were randomly selected to form the training set, and the remaining one-third formed the test set. To measure the recognition rate of the ALLRMM algorithm under different initial neighborhood values and dimensions, the initial neighborhood value k is assigned values of 2, 5, 10, 15, 20, and 25, and the dimensionality reduction dimension d is assigned values of 3, 5, 10, 15, and 20. The experimental results are shown in Figure 12.

As shown in Figure 12a, when the initial neighborhood value changes from 2 to 25, the recognition rate is negatively correlated with the initial neighborhood value, showing a downward trend. That is, a larger initial neighborhood value results in a lower recognition rate, which further demonstrates that the LLRMM algorithm improved by multi-manifold learning is appropriately sensitive to the selection of the initial neighborhood value. When changes from 3 to 20, the recognition rate is positively correlated with dimension. The figure shows that the average recognition rate of the improved LLRMM algorithm peaks at 97.50%. To further compare the influence of different initial neighborhood values on the algorithm, the LLE, LLRMM, and ALLRMM algorithms were selected for comparison under the ideal feature dimension of different initial neighborhood values. For the comparison, the average neighborhood values of all sample points under different initial neighborhood values were calculated after applying the adaptive neighborhood value iteration algorithm.

As shown in Table 5, the adaptive adjusted neighborhood value $k_{i}$ of the proposed ALLRMM algorithm approaches the ideal neighborhood value of 2. When the initial neighborhood value k is set to 20 and 25, it deviates considerably from its ideal value. However, the ALLRMM algorithm can also adjust the neighborhood value to 3 or 2, close to its ideal value. Clearly, the adaptive neighborhood value iteration algorithm is adequately stable and reliable. As shown in Figure 11 and Table 5, the proposed ALLRMM achieves the highest average recognition rate for various initial neighborhood values, whereas the LLE and ALLRMM achieve the highest average recognition rates of 90.75% and 97.5% only at ideal neighborhood values of k=15 and k=2, respectively. However, as shown in Figure 11, both the LLRMM and LLE are sensitive to the initial neighborhood value k. In contrast, even when the initial neighborhood value deviates significantly from its ideal value, ALLRMM continues to exhibit a good recognition rate.

**Figure 13:**
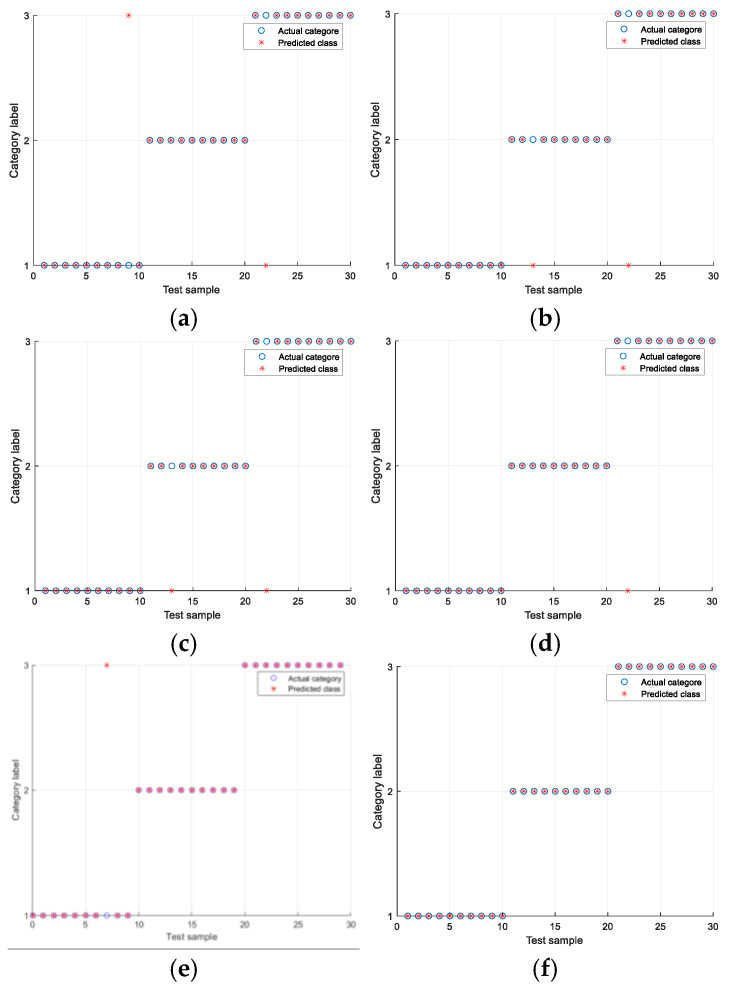
The highest recognition rate of different algorithms of set Ottawa-1: (**a**) Total features. (**b**) KPCA. (**c**) LLE. (**d**) LLRMM. (**e**) UMAP. (**f**) ALLRMM.

**Figure 14:**
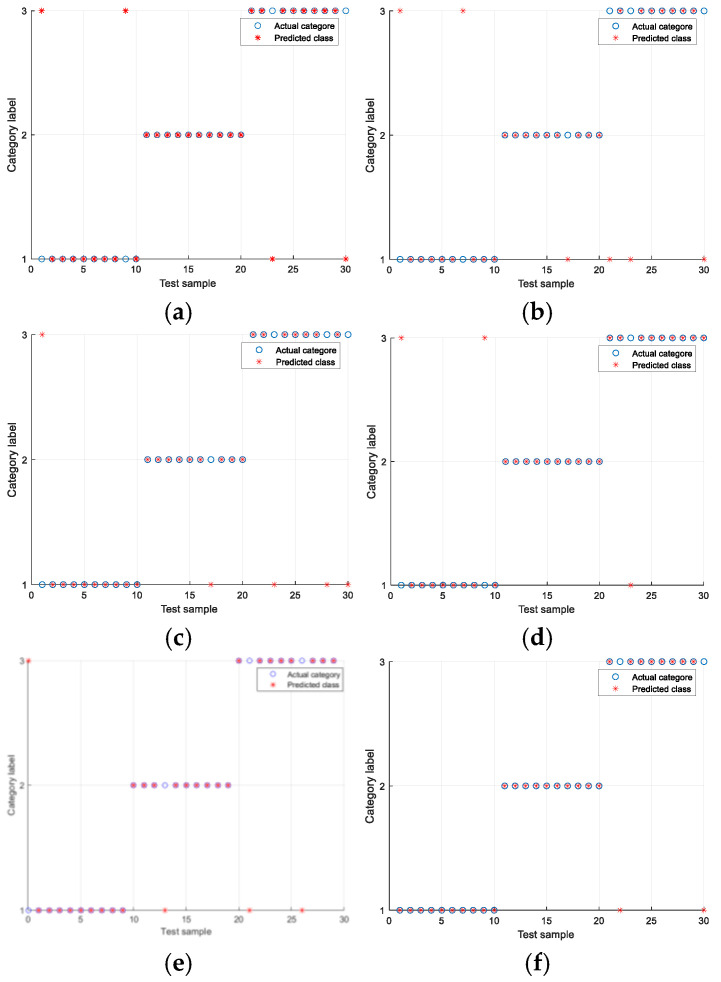
The highest recognition rate of different algorithms under four operating conditions of Ottawa-4 set: (**a**) Total features. (**b**) KPCA. (**c**) LLE. (**d**) LLRMM. (**e**) UMAP. (**f**) ALLRMM.

The experiments were conducted under the Ottawa Condition 1 and Condition 4 datasets. The experimental results are shown in Table 6 and Table 7, as well as Figure 13 and Figure 14. In the figures, the vertical axis represents the bearing health status, where Class 1 denotes the healthy condition, Class 2 indicates an inner race fault, and Class 3 corresponds to an outer race fault. The horizontal axis represents the test samples. As shown in Table 7, the fault diagnosis algorithm proposed based on multi-manifold learning ALLRMM has the same obvious effect under variable conditions. Under condition 1, the highest average recognition accuracy is 96.67%, and the highest recognition accuracy can reach 100%. As shown in Table 7, under working condition 4, the average recognition rate of the four algorithms is also the highest, which is 92.33%, and the highest recognition accuracy is 93.33%. Compared with KPCA and LLE, which are more sensitive to variable working condition data, the fault diagnosis accuracy is low. The algorithm based on ALLRMM proposed in this paper also has higher recognition accuracy under changing working conditions.

To further verify the superiority of the proposed ALLRMM algorithm, this paper conducts statistical significance and stability analyses. The statistical significance of the algorithm in improving recognition accuracy was evaluated using paired *t*-tests, while one-way ANOVA was employed to test the significance of differences in overall performance among various algorithms and to verify the reliability of the improvement results. The analysis results indicate that the accuracy improvement of the ALLRMM is statistically significant, and it exhibits low performance variance across multiple experiments, demonstrating good robustness and stability.

In summary, although mixed domain features can distinguish the running state of the rolling bearing well, the recognition rate remains low. The proposed algorithm can adjust the neighborhood value of each sample and bring it close to the ideal value, which reduces the sensitivity of conventional manifold learning algorithms to neighborhood value. In addition, it resolves the optimization problem and improves recognition accuracy by referring to the definition of manifold edge distance and the calculation of the manifold divergence matrix. Furthermore, the feature dimensionality is reduced to a certain extent after the features are extracted.

## 6. Conclusions

Considering its correlation to the selection of the nearest neighbor points, the sample density of each data point is used to calculate the density scaling factor, which is derived from the adaptive selection of the sample data neighborhood size. Subsequently, the within-manifold and between-manifold graphs are constructed adaptively, and the divergence matrix and edge distance of manifolds are defined. Finally, the feature extraction of the fault dataset is realized by maximizing the edge margin of the manifold and minimizing the within-class difference. The analysis results show the following: (1) The single feature in the time, frequency, and multiple fractal domains cannot completely distinguish the running state. In contrast, the mixed domain feature can better distinguish the running state; however, the recognition rate is not good enough. (2) The conventional learning method only preserves the local structural relationship between the data and does not directly contribute to data classification. Therefore, it cannot identify the distribution problem of multi-manifolds. (3) By improving the construction of manifold graphs through adaptive neighborhood selection, the sensitive problem of domain value selection of LLRMM is solved. Combining the definition of the manifold edge margin of LLRMM and the calculation of the manifold divergence matrix, an ALLRMM is proposed. The algorithm exhibits a remarkable dimensionality reduction effect and improved recognition accuracy.

Although the proposed ALLRMM shows significant improvement in fault diagnosis accuracy, particularly for complex manifold structures, certain limitations still exist. One notable limitation is its inability to recover manifolds with equidistant properties. This issue arises due to the algorithm’s focus on local structure preservation and edge margin processing, which inherently overlooks global equidistant relationships between data points.

Deep learning has been extensively applied in the field of fault diagnosis and has demonstrated remarkable efficacy. However, the manifold learning methodology adopted in this work fundamentally integrates linear projection with local structure preservation. Compared to deep learning approaches, it exhibits inherent limitations in representational capacity. Nevertheless, manifold learning offers distinct advantages in interpretability and model compactness, rendering it particularly suitable for resource-constrained industrial scenarios such as small-sample settings and edge computing. Future research may explore the integration of deep feature extraction with manifold margin discrimination mechanisms to further enhance accuracy while maintaining computational efficiency.

## Figures and Tables

**Table 1:** Ottawa dataset.

Bearing Health Conditions	Speed Varying Conditions			
	Increasing Speed	Decreasing Speed	Increasing Then Decreasing Speed	Decreasing then Increasing Speed
Healthy	H-A-1	H-B-1	H-C-1	H-D-1
	H-A-2	H-B-2	H-C-2	H-D-2
	H-A-3	H-B-3	H-C-3	H-D-3
Faulty(inner race fault)	I-A-1	I-B-1	I-C-1	I-D-1
	I-A-2	I-B-2	I-C-2	I-D-2
	I-A-3	I-B-3	I-C-3	I-D-3
Faulty(outer race fault)	O-A-1	O-B-1	O-C-1	O-D-1
	O-A-2	O-B-2	O-C-2	O-D-2
	O-A-3	O-B-3	O-C-3	O-D-3

**Table 2:** Statistical features in time domain.

Feature Name	Mathematical Formula	Feature Description
Mean value	$\bar{x}=\sum_{i=1}^{n}x_{i}/n$	Arithmetic average of signal amplitudes
Maximum amplitude	$x_{\max}=\underset{i=1,2,\ldots ,n}{\max}\{x_{i}\}$	Highest instantaneous value in the signal
Minimum amplitude	$x_{\min}=\underset{i=1,2,\ldots ,n}{\min}\{x_{i}\}$	Lowest instantaneous value in the signal
Peak-to-Peak value	$x_{p-p}=x_{\max}-x_{\min}$	Dynamic range of signal amplitudes
Root mean square	$x_{rmsv}=\sqrt{\sum_{i=1}^{n}x_{i}^{2}/n}$	Quadratic mean representing signal energy
Variance	$\sigma^{2}=\sum_{i=1}^{n}{\left(x_{i}-\bar{x}\right)}^{2}/n$	Measure of signal dispersion
Raw skewness	$\alpha =\sum_{i=0}^{n-1}x_{i}^{3}/n$	Unnormalized third moment for asymmetry
Raw kurtosis	$\beta =\sum_{i=0}^{n-1}x_{i}^{4}/n$	Unnormalized fourth moment for tail heaviness
Smoothness value	$x_{sra}={\left(\sum_{i=1}^{n}{\left|x_{i}\right|}^{1/2}/n\right)}^{2}$	Sensitivity to small amplitude variations
Normalized kurtosis	$K_{v}=\beta /{x_{rmsv}}^{4}$	Energy-compensated impulse detection
Normalized skewness	$x_{ds}=\alpha /\sigma^{3}$	Variance-normalized distribution asymmetry
Waveform factor	$x_{w}=(\frac{1}{n}\sum_{i=1}^{n}x_{i}^{2}{)}^{1/2}/\left(\frac{1}{n}\sum_{i=1}^{n}\left|x_{i}\right|\right)$	Ratio of RMS to mean absolute value
Crest factor	$x_{pi}=\underset{i=1,2,\ldots ,n}{\max}\left|x_{i}\right|/x_{rmsv}$	Peak-to-RMS ratio for impulse detection
Peak-to-Mean ratio	$x_{pf}=\underset{i=1,2,\ldots ,n}{\max}\left|x_{i}\right|/\bar{x}$	Peak amplitude relative to DC component
Smooth Peak ratio	$x_{m}=\underset{i=1,2,\ldots ,n}{\max}\left|x_{i}\right|/x_{sra}$	Peak normalized by smoothness value
Absolute mean	$\bar{X}=\left|\bar{x}\right|$	Magnitude of DC component

**Table 3:** Frequency-domain statistical characteristics.

Feature Name	Mathematical Formula	Feature Description
Spectral centroid	$f_{FC}=\sum_{i=2}^{n}\phi (i)x(i)/2\pi \sum_{i=1}^{n}x^{2}(i)$	Energy-weighted mean frequency, $\phi \left(i\right)$ denotes frequency weight of the *i*-th component
Mean square frequency	$f_{MSF}=\sum_{i=2}^{N}\phi^{2}(i)/4\pi^{2}\sum_{i=1}^{N}x^{2}(i)$	Second moment of spectral distribution
Root mean square frequency	$f_{RMSF}=\sqrt{f_{MSF}}$	Effective bandwidth measure
Frequency variance	$f_{VF}=f_{MSF}-{\left(f_{FC}\right)}^{2}$	Dispersion around spectral centroid
Spectral standard deviation	$f_{RFV}=\sqrt{f_{VF}}$	Standard deviation of frequency distribution

**Table 4:** Statistical characteristics of multiple fractal fields.

Feature Name	Mathematical Formula	Feature Description
Minimum singularity exponent	$a_{\min}$	Exponent for most singular regions
Maximum singularity exponent	$a_{\max}$	Exponent for smoothest regions
Multifractal spectrum width	$a_{0}$	Range of singularity strengths
Maximum fractal dimension	$f_{\max}=\max(f(a))$	Peak dimension in multifractal spectrum
Spectrum asymmetry	$\Delta f=f(a_{\max})-f(a_{\min})$	Asymmetry measure of singularity spectrum
Generalized Hurst exponent	h(q)	Scaling exponent function
Mean Hurst exponent	$\bar{h}=\sum_{i=1}^{N}h(i)/n$	Average scaling behavior
Hurst exponent range	$\Delta h(q)=h(q_{\min})-h(q_{\max})$	Multifractality strength indicator
Singularity exponent range	$\Delta a=a_{\max}-a_{\min}$	Width of singularity support

**Table 5:** Units for magnetic properties.

Initial Neighborhood Value		2	5	10	15	20	25
Rolling bearing fault dataset adaptive k value	LLE algorithm	2	5	10	15	20	25
	LLRMM algorithm	2	5	10	15	20	25
	ALLRMM algorithm	2	3	2	2	3	2

**Table 6:** Average identification accuracy in Ottawa dataset 1.

Method	Average Recognition Rate
Total features	91.67%
LLE	90.00%
KPCA	92.00%
UMAP	93.30%
LLRMM	93.33%
ALLRMM	96.67%

**Table 7:** Average recognition rate of different algorithms in Ottawa dataset 4.

Method	Average Recognition Rate
Total features	86.67%
KPCA	71.33%
UMAP	76.65%
LLE	81.00%
LLRMM	89.33%
ALLRMM	92.33%

## Data Availability

The data presented in this study are not publicly available due to ongoing project status and confidentiality restrictions imposed by the industrial partner. The dataset contains proprietary product parameters that are protected under a non-disclosure agreement. Access to the data may be granted on reasonable request, subject to written permission from the manufacturer. Requests should be directed to the corresponding author.
